# Screening of Potential Angiotensin-Converting Enzyme-Inhibitory Peptides in Squid (*Todarodes pacificus*) Skin Hydrolysates: Preliminary Study of Its Mechanism of Inhibition

**DOI:** 10.3390/md23020081

**Published:** 2025-02-13

**Authors:** Mingyuan Li, Qianqian Liang, Yurui Zhang, Xin Jiang, Yuan Gu, Xin Song, Xichang Wang, Wenzheng Shi

**Affiliations:** 1 College of Food Sciences & Technology, Shanghai Ocean University, Shanghai 201306, China; 15541645317@163.com (M.L.); lqq18152566062@163.com (Q.L.); 13162885465@163.com (Y.Z.); 13053523375@163.com (X.J.); xcwang@shou.edu.cn (X.W.); 2 Dandong Yuanyi Seafood Finishing Products Co., Ltd., Dandong 118300, China; guyuan@ddyyhc.com (Y.G.); songxin@ddyyhc.com (X.S.)

**Keywords:** squid skin, ACEI peptide, sequence identification, molecular docking

## Abstract

Background: Hypertension has been identified as a significant risk factor for cardiovascular disease. Given the prevalence of the adverse effects of angiotensin-converting enzyme-inhibitory (ACEI) drugs, natural and effective alternatives to these medications need to be identified. Methods: An investigative study was conducted to assess the ACEI capacity and structural characteristics of enzymatic hydrolysates with varying molecular weights derived from squid skin. The amino acid sequences of the enzymatic digests were analyzed via Nano LC-MS/MS and screened for peptides with ACEI activity using an in silico analysis. Furthermore, molecular docking was employed to investigate the interaction between potential ACEI peptides and ACE. Results: TPSH-V (MW < 1 kDa) exhibited the highest rate of ACEI, a property attributable to its substantial hydrophobic amino acid content. Additionally, TPSH-V exhibited high temperature and pH stability, indicative of regular ordering in its secondary structure. The binding modes of four potential novel ACEI peptides to ACE were predicted via molecular docking with the sequences of FHGLPAK, IIAPPERKY, RGLPAYE, and VPSDVEF, all of which can bind to the ACE active site via hydrogen bonding, with FHGLPAK, RGLPAYE, and VPSDVEF being able to coordinate with Zn^2+^. Conclusions: Squid skin constitutes a viable resource for the production of ACEI peptides.

## 1. Introduction

*Todarodes pacificus* is a type of squid that is popular around the world for its nutritional benefits [1]. In China, the meat and legs are the main economic products of squid [2], while the skin, viscera, and other scraps are generally processed into fishmeal or disposed of via burial, resulting in environmental pollution and a waste of resources [3]. Squid skin accounts for about 8–13% [4] of the total weight of squid. Compared with by-products such as squid guts and eyes, squid skin has high crude protein content and is rich in essential amino acid content that is easily absorbed and used by the human body when the squid skin protein is hydrolyzed into small peptides [5]. Currently, bioactive peptides such as antioxidant peptides [6], antifreeze peptides [7], DPP-IV inhibitory peptides [8], and ACEI peptides have already been extracted from squid skin.

Hypertension is a major cause of cardiovascular disease and premature deaths globally. The worldwide prevalence is on the rise due to the aging population and increased risk factors such as unhealthy lifestyles [9]. At present, ACEIs in antihypertensive drugs are known as first-line drugs for treating hypertension. They play an antihypertensive role by reducing the formation of Ang II and inhibiting the degradation of bradykinin [10]. However, this traditional chemically synthesized drug has many side effects, for instance, cough, allergies, taste disorders, and skin rashes [11]. Food-derived antihypertensive peptides have gradually gained widespread attention because of their widespread sources, high safety, and absence of side effects. Nakamura et al. [12,13] found that the tripeptides VPP (IC_50_ = 9.0 μM) and IPP (IC_50_ = 5.0 μM) could be obtained from sour milk. VPP and IPP are currently commercialized ACEI peptides. Although the immediate inhibitory effect is lower than that of synthetic drugs such as captopril (IC_50_ = 0.02 μM) [14], ACEI peptides have a more modest antihypertensive effect [15]. Through molecular docking, Chamata et al. [16] found that the whey hydrolysates IIAE and LIVTQ interacted with the amino acid residues Asp 140 and Ala 332 of ACE, respectively, which are the same as those of lenopril, suggesting that natural peptides may be effective ACEIs.

ACEI peptides come from a wide range of sources. Xu et al. [17] identified six novel peptides in soybean isolate protein hydrolysate, including IY, YVVF, LVF, WMY, LVLL, and FF. Auwal et al. [18] identified five new peptides consisting of five to seven amino acids in stone fish protein hydrolysates and, through molecular docking, found that the peptides exerted antihypertensive effects through hydrogen bonding and electrostatic interactions to form complexes with ACE and destabilize the stable structure of ACE. The oceans cover more than 70% of the Earth’s surface [19], and their organisms are a rich source of quality proteins. With the increasing number of fish caught, the use of by-products has attracted a great deal of attention. Studies have been conducted on the preparation of ACEI peptides in by-products such as Alaska pollack skin [20] and shortfin scad skin [21]. However, most studies have been conducted on hydrolysates of collagen [22] and gelatin [23,24] produced from fish skin, which is a cumbersome process and utilizes an incomplete range of proteins.

In this study, squid skin enriched in hydrophobic amino acids (HAAs) was directly hydrolyzed, the activity and spatial structure of different molecular weight peptides in the hydrolysates were determined, and the ACEI peptide and its action mechanism were explored. This work not only provides new insights into the preparation of novel ACEI peptides but also increases the utilization of squid by-products.

## 2. Results and Discussion

### 2.1. Amino Acid Analysis of ACE Inhibitory Peptides with Different Molecular Weights (MWs)

Amino acid composition is an important factor affecting the properties of bioactive peptides [25]. In this study, the amino acid composition and content of ACEI peptides with different molecular weights after ultrafiltration were analyzed and compared with *Todarodes pacificus* skin hydrolysates (TPSHs). As shown in Table 1, 17 amino acids were detected, including 11 non-essential amino acids and 6 essential amino acids. In addition, after ultrafiltration, the content of branched-chain amino acids (BCAAs) increased with decreasing molecular weight. This increase was positively correlated with ACEI activity, suggesting that valine (Val), methionine (Met), and isoleucine (Ile) contributed to the enhancement of ACEI activity [26]. Moreover, the HAAs of TPSH-Ⅰ had the lowest content, at 10.08 ± 0.38 g/100 g, and TPSH-V had the highest content of HAAs, at 22.70 ± 0.14 g/100 g, results similar to the findings of Kumar [27]. Some studies have shown that the presence of HAAs, BCAAs, and aromatic amino acids (AAAs) could enhance the ACEI activity of the peptide [28,29,30]. The ACEI activity and the content of HAAs and BCAAs in TPSH-V were significantly higher than those in other groups (*p* < 0.05), which indicates that ultrafiltration is an effective separation and purification method.

### 2.2. ACE Inhibitory Activity with Different Molecular Weights

**Figure 1:**
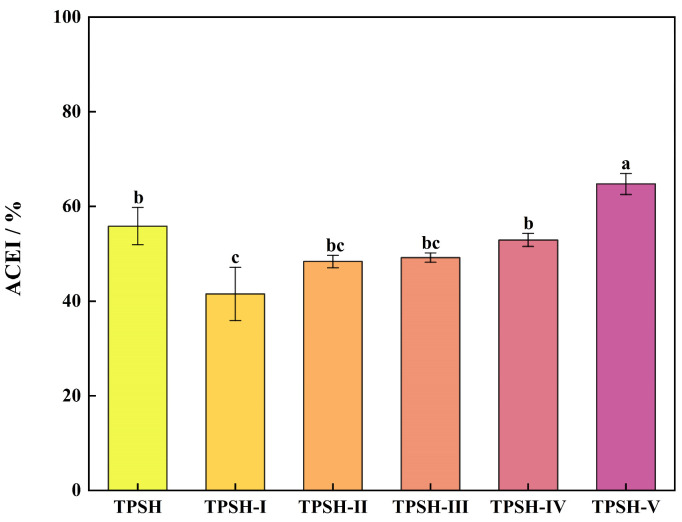
ACEI rates of peptides with different molecular weights. Values are expressed as mean ± SD (*n* = 3). Different letters represent significant differences from each other (*p* < 0.05), and the same letters mean not significant (*p* > 0.05).

The ACEI rates of TPSH and the five fractions obtained via ultrafiltration are shown in Figure 1. TPSH-V had the highest ACEI rate of 64.76 ± 2.22%; TPSH-I had the lowest ACEI rate of 41.51 ± 5.63%, which might be due to the sequestration of macromolecules such as pigments and a small number of aggregated peptides with ACEI activity [31,32]. These rates are consistent with the findings of Lee et al. [33]. The ACEI rate of TPSH-V differed significantly from that of TPSH (*p* < 0.05), indicating that ultrafiltration separation was an effective method for separating highly inhibited components. Moreover, the IC_50_ of the TPSH-V was 0.70 mg/mL. The IC_50_ value of the protein hydrolysates of sardinelle by-products was reported to be 0.81 mg/mL [34]. The IC_50_ value of the enzymatic hydrolysate (<1 kDa) of squid by-products separated using RP-HPLC was 1.34 mg/mL [35]; all of their inhibitory effects on ACE were less impactful than those of TPSH-V. These findings suggest that TPSH-V contains highly active ACEI peptides, as well as that squid skins are an excellent source of ACEI peptides.

In summary, in order to further study the structure–function relationship of ACEI peptides, TPSH-V fractions were collected and further analyzed in this study.

### 2.3. Secondary Structure of TPSH and TPSH-V

**Figure 2:**
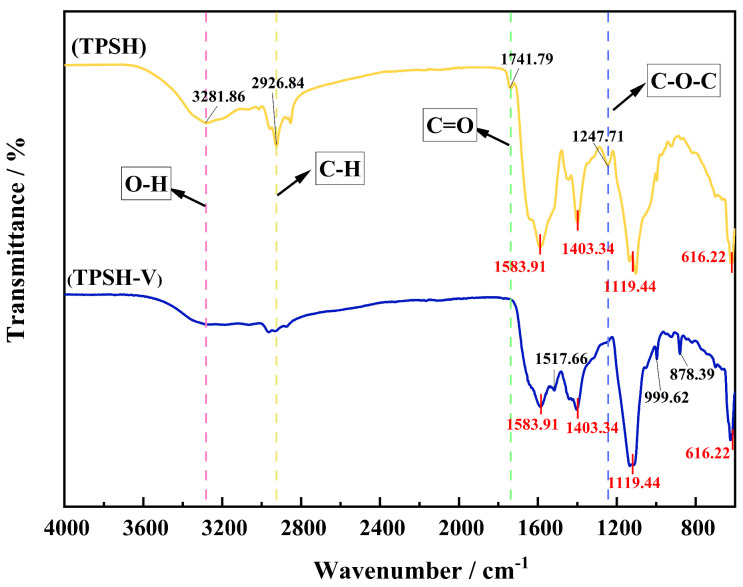
The FTIR spectra of TPSH and TPSH-V.

A Fourier transform infrared spectrometer (FTIR spectrometer) is a tool for analyzing the secondary structure of peptides based on the absorption wavelengths and intensities of the molecule [36]. As shown in Figure 2, functional groups such as O-H (3300~2500 cm^−1^), saturated C-H (3000~2800 cm^−1^), -C=O (1850~1600 cm^−1^), and C-N (1360~1180 cm^−1^) disappeared or weakened in TPSH-V, while C=C (1610~1370 cm^−1^), -CHO (955~1058 cm^−1^), and aromatic ring (900~690 cm^−1^) appeared [37,38,39]. In addition, the amide I band (1600~1700 cm^−1^) of TPSH-V consisted of 21.78 ± 0.04% β-sheets, 13.46 ± 0.02% random coils, 15.04 ± 0.07% α-helices, and 49.71 ± 0.06% β-turns. The changes in functional groups in TPSH-V were caused by the variation in the spatial structure of the peptide, which resulted from differences in the number of amino acids [40]. The content of amino acids in TPSH and TPSH-V was speculated to be different, resulting in the exposure of certain reactive groups and different ACEI activities. In addition, the relative contents of β-turns and β-sheets were relatively large in the amide I band of TPSH-V, which could suggest that β-turn and β-sheet play important roles in promoting ACEI activity [41]. Moreover, the more β-turns, the more ordered and regular the secondary structure, thus indicating that the peptide had a stable structure [42]. This was consistent with the structure of TPSH-V.

### 2.4. TPSH-V Stability Analysis

Peptides are structurally unstable and easily destroyed by some factors, leading to low bioavailability and inhibiting development [43]. At present, pH and temperature stability are of the greatest concern and are also the main evaluation indexes of analog peptide processing treatments [44]. Based on this, the effects of temperature and pH on the stability of potential ACEI peptides in TPSH-V were investigated in this study.

**Figure 3:**
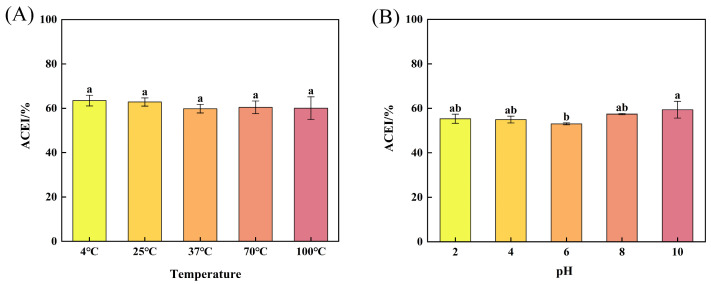
Effect of temperature (**A**) and pH (**B**) on the activity of ACEI peptide in TPSH-V. Values are expressed as mean ± SD (*n* = 3). Different letters represent significant differences from each other (*p* < 0.05), and the same letters mean not significant (*p* > 0.05).

The ACEI rates at different temperatures are shown in Figure 3A. No significant difference was found in the ACEI rates of the peptides treated with different temperatures (*p* > 0.05), which suggests that the peptides had good temperature stability. This result was consistent with the results from the study of Li et al. [45] and support the choice of sterilization method for ACEI peptide products.

The ACEI rates at different pH values are shown in Figure 3B. No significant difference was found in the ACEI rates at pH 2, 4, 8, and 10 (*p* > 0.05), but the lowest ACEI activity (53.00 ± 0.44%) was exhibited at pH 6. This might be because the pH of 6 is close to the isoelectric point [46], when the structural contraction of proteins lead to aggregation of the protein molecules, thereby affecting the interactions between the peptides and ACE, leading to a decrease in ACEI activity. This trend was analogous to the results of Zheng et al. [47].

### 2.5. Peptide Identification

**Figure 4:**
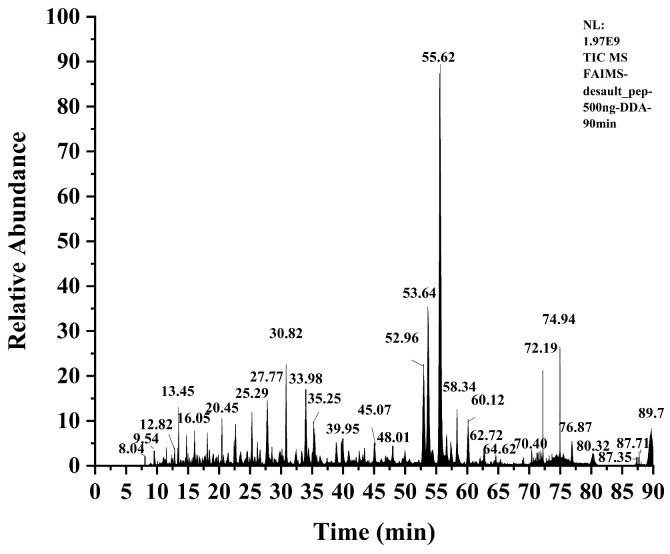
Total ion flow diagram of TPSH-V.

According to the previous speculation, TPSH-V should contain highly active ACEI peptides, and to verify this speculation, Nano LC-MS/MS was used to probe the amino acid sequences of the peptides. A total ion flow diagram of TPSH-V is shown in Figure 4. Furthermore, seventeen peptides were screened, and their toxicity and ACEI activities are shown in Table 2.

The seventeen identified peptides were imported into the AHTpin platform, in which the FHGLPAK (Phe-His-Gly-Leu-Pro-Ala-Lys), IIAPPERKY (IIe-Ile-Ala-Pro-Pro-Glu-Arg-Lys-Tyr), RGLPAYE (Arg-Gly-Leu-Pro-Ala-Tyr-Glu), and VPSDVEF (Val-Pro-Ser-Asp-Val-Glu-Phe) peptides all had AHT-SVM scores greater than 0. The FHGLPAK, IIAPPERKYSVM, and VPSDVEF scores were greater than 1, so FHGLPAK, IIAPPERKY, RGLPAYE, and VPSDVEF were assumed to have ACEI capabilities [48]. A comparison with ACEI peptides recorded in the BIOPEP-UWM database [49] revealed that none of these four peptides have been studied in terms of ACEI capabilities and could be investigated as potential novel ACEI peptides.

ACEI peptides are typically short peptides composed of 2 to 12 amino acids, and the amino acid species at the peptide terminals have a great influence on the ACEI activity [50]. To investigate the structural features of the ACEI peptide, the amino acid sequences of 1184 ACEI peptides in the BIOPEP-UWM database were analyzed.

Research studies shown that the top three frequencies of N-terminal occurrence were Leu (13.01%), Val (10.05%), and Ile (8.61%), indicating that the N-terminal end with HAAs was more likely to be ACEI peptides, which was in agreement with that suggested by Iwaniak et al. [51]. In addition, Hao et al. [52] considered that HAAs at the N-terminal end enhanced the binding efficiency of peptides to the ACE active center, thus enhancing the ACEI ability of the peptide. Furthermore, the frequency of amino acids at the C-terminus was polarized, with the most frequent of these being Pro (24.58%), followed by Tyr (10.30%), Lys (7.52%), Leu (7.52%), Arg (7.18%), and Phe (7.09%), all with frequencies of more than 5%. Ding et al. [30] showed that when Pro appeared at the C-terminus, it had a higher ACEI activity than when it appeared in the penultimate position and that its presence caused the peptide to exhibit higher in vitro digestive stability [53]. In addition, the presence of HAAs and AAAs may enhance the ACEI of the peptide [54,55]. Abdelhedi et al. [56] demonstrated that positively charged Lys and Arg located at the C-terminal end also contributed to the enhancement of ACE inhibitory activity.

The amino acids located at the N-terminal and C-terminal ends of IIAPPERKY were Ile and Tyr, respectively; the amino acids located at the N-terminal and C-terminal ends of VPSDVEF were Val and Phe, respectively; and the amino acid located at the C-terminal end of FHGLPAK was Lys, which resulted in AHT-SVM scores greater than 1 for these three peptides. Interestingly, these amino acids located at the N-terminal and C-terminal ends of the three peptide chains were HAAs, and substances with high HAA contents were speculated to contain potential ACEI peptides. The penultimate amino acid at the C-terminal end of RGLPAYE was Tyr, which also conforms to the structural characteristics of ACEI peptides. Therefore, the mechanism of action of the four potential ACEI peptides mentioned above in relation to ACE were investigated.

### 2.6. Molecular Docking Analysis of Four Potential Novel ACEI Peptides Bound to ACE

To further reveal the mechanism of action of ACEI peptides, pre-treated FHGLPAK, IIAPPERKY, RGLPAYE, and VPSDVEF were semi-flexibly docked with ACE using the Discovery Studio 2024 Client software (DS). The “-CDOCKER Energy” value was used to evaluate the degree of binding between the receptor and the ligand in different conformations: the higher the “-CDOCKER Energy” value, the more tightly bound the ligand and the receptor protein [57,58]. The results showed that the “-CDOCKER Energy” values of FHGLPAK, IIAPPERKY, RGLPAYE, and VPSDVEF with ACE were 131.702, 112.44, 117.731, and 125.667 kcal/mol, respectively, indicating that these four ACEI peptides could bind to ACE at this binding site to form a stable complex. Furthermore, the root mean square deviation (RMSD) values of 1.11 Å, 1.88 Å, 1.32 Å, and 1.20 Å, respectively, indicated the reliability of the molecular docking method employed.

ACE had three major active site pockets: S1, S2, and S1’. Ala-354, Glu-384, and Tyr-523 were found at S1; Gln-281, His-353, Lys-511, His-513, and Tyr-520 were present at S2; and Glu-162 was seen at S1’. In addition, the active center of ACE, Zn^2+^ (Zn-701), coordinated binding to His-383, His-387, and Glu-411 to form a tetrahedral structure, which plays a crucial role in stabilization [59]. Therefore, the interaction with the above amino acid residues was considered to be the principle mechanism behind inhibiting ACE activity [60].

**Figure 5:**
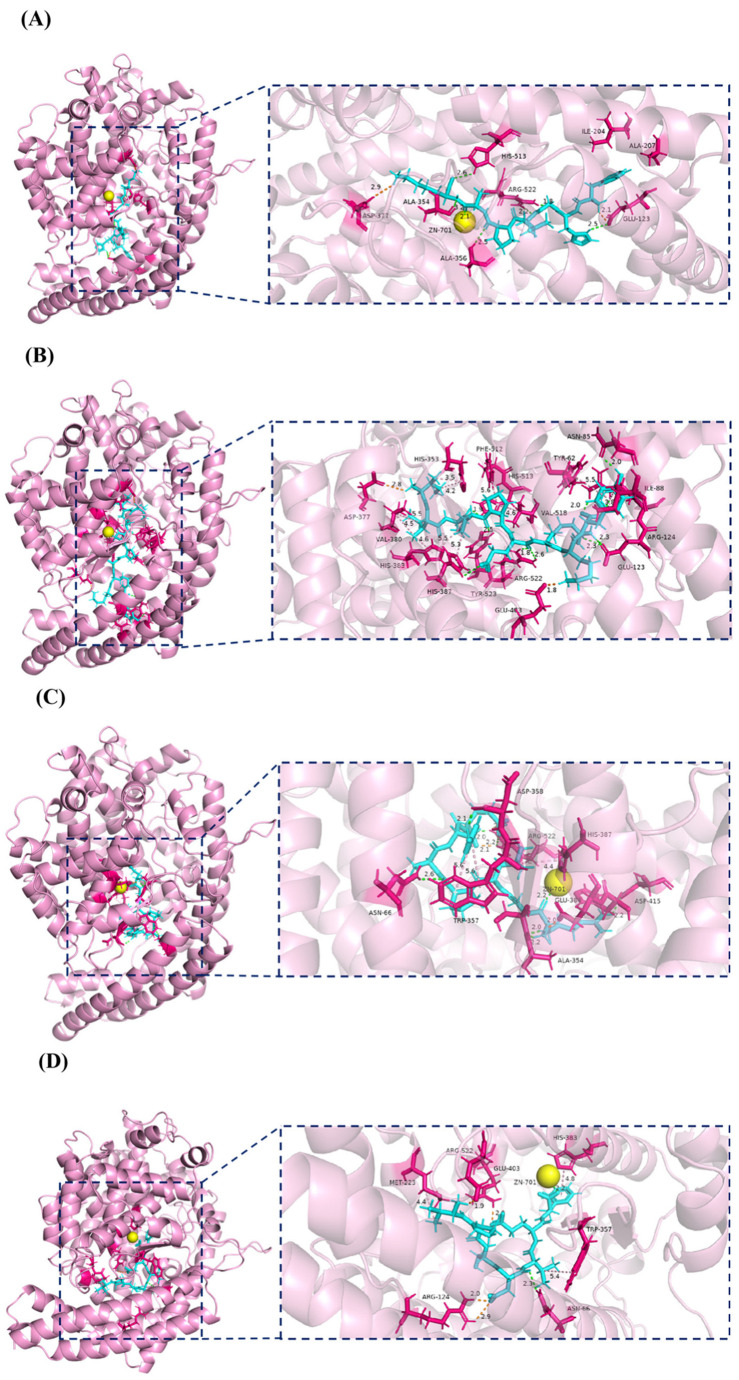
(**A**–**D**) Three-dimensional diagrams of the docking results of FHGLPAK, IIAPPERKY, RGLPAYE, and VPSDVEF with ACE, where the blue sticks indicate the ligands and the pink sticks indicate the residues of ACE. Their 2D diagrams are shown in the Supplementary Materials.

It was proved that, the more the peptide interacted with amino acids in the S1, S2, S1’, and zinc-binding domains of ACE, the stronger the destructive force on the ACE structure and, thus, the higher the ACEI activity of the peptide [61]. As shown in Figure 5, FHGLPAK was able to interact with Ala354 in the S1 pocket, His-513 in the S2 pocket, and the Zn^2+^ active center. IIAPPERKY interacted with Tyr-523 in the S1 pocket and His-353 in the S2 pocket, and His383 in the zinc-binding domain and bound tightly to His-383 and His-387, resulting in a distortion of the tetrahedral ligand and disrupting the ACE stability [62], thus achieving the effect of inhibition. RGLPAYE interacted with Ala-354, Glu-384 in the S1 pocket, the Zn^2+^ active center, and His-383 in the zinc-binding domain. VPSDVEF interacted with the Zn^2+^ active center and His-383 in the zinc-binding domain.

Furthermore, the inhibitory effect of ACE was mainly achieved through hydrogen bonding interactions and Zn^2+^ interactions between the peptide and ACE [63]. FHGLPAK, RGLPAYE, and VPSDVEF were all able to bind to the Zn^2+^ active center. Moreover, FHGLPAK formed hydrogen bonds with Glu-123, Arg-522, His-513 (S2 pocket), Ala-354 (S1 pocket), and Ala-356; IIAPPERKY formed hydrogen bonds with His-387 (zinc ligand), His-513 (S2 pocket), Tyr-523 (S1 pocket), Arg-522, Glu-123, Asn-85, and Arg-124; RGLPAYE formed hydrogen bonds with Asp-415, Ala-354 (S1 pocket), Asp-358, Asn-66, and Arg-522; and VPSDVEF formed hydrogen bonds with Asn-66. These results suggest that the four peptides—FHGLPAK, IIAPPERKY, RGLPAYE, and VPSDVEF—have potential ACEI abilities.

## 3. Materials and Methods

### 3.1. Materials

Squid (*Todarodes pacificus*) skin was obtained from Dandong Yuanyi Seafood Finishing Products Co., Ltd. (Dandong, Liaoning, China). Alkaline protease (120 U/mg) was provided by Shanghai Yuanye Biotechnology Co., Ltd. (Shanghai, China). HEPES (N-2-hydroxyethylpiperazine-N′-2-ethanesulfonic acid, 99% biotechnology grade) was provided by Shanghai McLean Biochemical Science and Technology Co., Ltd. (Shanghai, China). Formic acid (FA), chromatography-grade acetonitrile (ACN), ACE (0.1 U-from rabbit lung), and FAPGG (N-[3-(2-furanyl)acryloyl]-Phe-bis-glycine) were from Sigma-Aldrich Chemicals Company (St. Louis, MO, USA). The water used for the experiments was ultrapure water.

### 3.2. Preparation and Ultrafiltration of TPSH

TPSH was prepared as follows: the squid skin was treated with isopropanol solution (15 *v*/*v*) as the degreasing reagent at 1:15 (*w*/*v*) for 24 h to obtain degreased squid skin. Ultrapure water was added into the defatted squid skin at the ratio of 1:7.8 (*w*/*v*) and heated at 90 °C for 15 min to remove the effect of endogenous enzymes. Upon cooling, the enzymatic hydrolysate was homogenized, and the pH was adjusted to 10. Alkaline protease was added according to the enzyme amount of 6190 U/g, and the reaction was placed in a magnetic stirrer at 40 °C for 3.11 h. After hydrolysis, the enzyme was inactivated using boiling water; then, it was cooled and centrifuged at 10,000 r/min for 20 min, and the supernatant was filtered.

Ultra centrifugal filters were used to separate substances of different molecular weights. After washing with ultrapure water, TPSH was added into the 10 kDa ultra centrifugal filter, centrifuged for 30 min at 6000 r/min to collect the lower layer of liquid. Centrifugation was continued in the 5 kDa ultra centrifugal filter, and so on, to obtain a molecular weight greater than 10 kDa: TPSH-I; molecular weight of 5~10 kDa: TPSH-II; molecular weight of 3~5 kDa: TPSH-III; molecular weight of 1~3 kDa: TPSH-IV; molecular weight less than 1 kDa: TPSH-V.

TPSH and the five fractions obtained by ultrafiltration were prepared at a concentration of 2 mg/mL after lyophilization and their ACEI rates were determined.

### 3.3. Determination of Total Amino Acids

The amino acid composition and content of TPSH, TPSH-I, TPSH-II, TPSH-III, TPSH-IV, and TPSH-V were determined using an automatic amino acid analyzer with reference to the method of Yao et al. [64].

### 3.4. In Vitro ACEI Activity Assay

The ACEI rate was determined according to a previous method [65]. Using FAPGG as a substrate for ACE action, it was hydrolyzed to FAP (FA-Phe) and GG (Gly-Gly). Then, 10 µL of ACE (0.1 U/mL) and 50 µL of FAPGG (1 mM) were added to the 96-well plate, 40 µL of ultrapure water was added to the blank group, and 40 µL of the sample was added to the sample group. Then, the change in absorbance at 340 nm before and after 30 min at 37 °C was recorded. The change in the absorbance value of the blank was recorded as *A*, and the change in the absorbance value of the sample was recorded as *B*. The formula for ACEI rate is as follows:

(1) $$ACEI\;rate\;\left(\%\right)=\frac{A-B}{A}\times 100\;\%$$

The IC_50_ value is the concentration of the sample when the ACEI rate is at 50%. The samples were set to five mass concentrations: 2.00, 1.00, 0.50, 0.10, and 0.05 mg/mL. The ACEI rates of the different groups were determined, and the results were entered into GraphPad software (GraphPad Prism 8.0.2) to obtain the IC_50_ values after fitting the curve.

### 3.5. Determination of Peptides’ Secondary Structure Using Fourier Transform Infrared Spectroscopy

Following the method used by Jiang et al. [66] with minor modifications, the FTIR of the peptide samples was analyzed using an FTIR imaging system (Spotlight 400, PerkinElmer, Seer Green, UK). The collagen samples were scanned 32 times in the spectral range of 4000–600 cm^−1^ with a resolution of 4 cm^−1^. OMNIC was used to map and select 1700~1600 cm^−1^ amide I bands in the selection map; then, Peak Fit 4.12 software was used for the analysis.

### 3.6. Temperature Stability Analysis

TPSH-V lyophilized powder was configured into a 2 mg/mL solution with ultrapure water and left at 4, 25, 37, 75, and 100 °C for 2 h. The ACEI rate was determined after rapid cooling.

### 3.7. pH Stability Analysis

TPSH-V lyophilized powder was configured into a 2 mg/mL solution with ultrapure water; the pH was adjusted to 2, 4, 6, 8, and 10 with NaOH and HCl; then, the solution was left at 4 °C for 2 h (adjusting the pH every 30 min). Finally, the ACEI rate was determined after adjusting the pH to 8.2.

### 3.8. Identification of the Sequence of TPSH-V via Nano LC-MS/MS

TPSH-V samples were desalted and lyophilized on a sep-Pak C18 desalting column (Waters Co., Ltd., Milford, MA, USA). The chromatographic conditions were as follows: column, Thermo Scientific UltiMate 3000-C18 (Waltham, MA, USA) (200 mm × 75 mm, 1.9 μM); injection volume, 5 μL; flow rate, 4 μL/min; mobile phase A, water + 0.1% FA; and mobile phase B, 80% ACN + 0.1% FA. The liquid phase was eluted with a 90 min elution gradient, and the specific parameters of the assay elution conditions are shown in Table 3. The mass spectrometry conditions are shown in Table 4. The screening parameters are shown in Table 5.

The Uniprot-SwissProt database (https://www.uniprot.org (accessed on 30 November 2024)) was used to analyze and identify the peptides from squid (*Todarodes pacificus*) and to retrieve the resulting spectra and peptides.

### 3.9. Peptide In Silico Screening

The peptide sequences screened via LC-MS/MS were searched and compared with the ACEI peptide sequences and structures recorded in the BIOPEP-UWM (https://biochemia.uwm.edu.pl/biopep/start_biopep.php (accessed on 30 November 2024)) bioactive peptide database. Referring to the method of Zhang et al. [67], the ACEI peptide sequences were further screened using the AHTpin platform (https://webs.iiitd.edu.in/raghava/ahtpin/ (accessed on 30 November 2024)) and the ToxinPred platform (https://webs.iiitd.edu.in/raghava/toxinpred/design.php (accessed on 30 November 2024)) for unreported ACE peptides.

### 3.10. Molecular Docking Between Four Novel Peptides and ACE

The crystal structure of ACE (PDBID: 1O86) was downloaded from the RCSB PDB database [68]. Four small molecules were obtained by entering the peptide sequences in “Specify” of the Discovery Studio 2024 Client software (DS). ACE and four peptides were processed using the “Prepare Protein” and “Full Minimization” functions, respectively, in DS. Then, Zn^2+^ was set as the active center; the sphere radius was 15 Å; and the x, y, and x coordinates were 43.821, 38.240, and 46.712, respectively. In addition, the force field of the whole process was “CHARMm”, the “Ionization Method” in “Prepare Protein” was “pH based”, and the “Algorithm” in “Energy minimizer” was “Smart Minimizer”. The CDOCKER module was then selected for molecular docking, with “Pose Cluster Radius” set to 0.5 to ensure that the docked conformations were as diverse as possible, and the rest of the parameters were left on their default values. Finally, the obtained images were processed, and the results were analyzed using DS (BIOVIA Discovery Studio 2024) and PyMOL software (PyMOL 2.5.7. DeLano Scientific LLC, Palo Alto, CA, USA).

### 3.11. Statistical Analysis

The determination of all samples was repeated three times. Levene’s test and a Shapiro–Wilk test were used to evaluate the homoscedasticity (*p* > 0.05) and normal distribution of the data (*p* > 0.05), respectively. Subsequently, significant differences (*p* < 0.05) were evaluated using a test of variance and Duncan’s multiple range test, and the calculations were performed using SPSS statistical software (26.0 version, Michigan State University, East Lansing, MI, USA).

## 4. Conclusions

In this study, four potential novel ACEI peptides were obtained from *Todarodes pacificus* skin hydrolysates. The squid skin was hydrolyzed with alkaline protease, and the squid skin hydrolysate was separated into five fractions of ACEIs with different molecular weights via ultrafiltration. Fractions with molecular weights less than 1 kDa had the strongest ACEI activity and the most stable structure. From the analysis of the amino acid composition and content of this fraction of peptides, the amino acid sequence and the ACEI mechanism, the high content of HAAs suggested that the hydrophobicity of the amino acid residues had a higher effect on ACEI activity. In addition, four potential novel ACEI peptides were identified: FHGLPAK, IIAPPERKY, RGLPAYE, and VPSDVEF. The inhibition mechanism of the molecular docking simulation showed that all four peptides were tightly bound to the ACE active site, which proved that they all had potential ACEI ability. In conclusion, squid skin is a good source of ACEI peptides and can be added to beverages or foods to help control hypertension.

## Figures and Tables

**Table 1:** Amino acid composition and content.

Amino Acid	Content (g/100 g)					
	TPSH	TPSH-Ⅰ	TPSH-Ⅱ	TPSH-Ⅲ	TPSH-Ⅳ	TPSH-Ⅴ
Asp	5.14 ± 0.35 ^d^	3.80 ± 0.16 ^e^	4.10 ± 0.05 ^e^	7.87 ± 0.09 ^a^	5.63 ± 0.12 ^c^	7.13 ± 0.16 ^b^
Thr ^#^	1.98 ± 0.08 ^c^	1.23 ± 0.06 ^d^	1.96 ± 0.02 ^c^	2.48 ± 0.00 ^a^	2.27 ± 0.08 ^b^	2.54 ± 0.04 ^a^
Ser	2.32 ± 0.13 ^c^	1.47 ± 0.04 ^d^	2.69 ± 0.04 ^b^	2.77 ± 0.05 ^b^	2.81 ± 0.08 ^b^	2.98 ± 0.02 ^a^
Glu	6.51 ± 0.39 ^c^	4.13 ± 0.03 ^e^	5.93 ± 0.05 ^d^	8.81 ± 0.10 ^a^	7.68 ± 0.27 ^b^	8.70 ± 0.28 ^a^
Gly	5.64 ± 0.61 ^c^	3.73 ± 0.11 ^d^	5.88 ± 0.07 ^c^	8.75 ± 0.10 ^a^	7.36 ± 0.26 ^b^	8.77 ± 0.24 ^a^
Ala *	3.35 ± 0.20 ^c^	1.95 ± 0.04 ^d^	4.32 ± 0.07 ^b^	4.24 ± 0.03 ^b^	4.34 ± 0.11 ^b^	4.65 ± 0.14 ^a^
Cys	0.21 ± 0.05 ^b^	0.15 ± 0.01 ^b^	0.30 ± 0.00 ^a^	0.16 ± 0.05 ^b^	0.16 ± 0.00 ^b^	0.20 ± 0.00 ^b^
Val *^#1^	2.23 ± 0.09 ^c^	1.27 ± 0.09 ^d^	2.37 ± 0.02 ^c^	2.62 ± 0.05 ^b^	2.60 ± 0.07 ^b^	2.85 ± 0.08 ^a^
Met *^#1^	1.24 ± 0.01 ^a^	0.46 ± 0.19 ^b^	0.08 ± 0.02 ^c^	0.52 ± 0.20 ^b^	0.65 ± 0.08 ^b^	1.22 ± 0.23 ^a^
Ile *^#1^	1.98 ± 0.09 ^c^	1.19 ± 0.08 ^d^	1.97 ± 0.03 ^c^	2.33 ± 0.05 ^ab^	2.26 ± 0.07 ^b^	2.49 ± 0.08 ^a^
Leu *^#^	3.17 ± 0.15 ^b^	1.79 ± 0.09 ^c^	3.83 ± 0.07 ^a^	3.40 ± 0.05 ^b^	3.72 ± 0.10 ^a^	3.81 ± 0.08 ^a^
Tyr ^2^	1.88 ± 0.20 ^b^	1.08 ± 0.04 ^c^	2.01 ± 0.05 ^ab^	2.09 ± 0.05 ^ab^	2.11 ± 0.04 ^ab^	2.23 ± 0.04 ^a^
Phe *^#2^	2.23 ± 0.16 ^d^	1.20 ± 0.06 ^e^	3.00 ± 0.05 ^a^	2.39 ± 0.09 ^cd^	2.61 ± 0.05 ^bc^	2.63 ± 0.06 ^b^
Lys	2.81 ± 0.09 ^c^	1.45 ± 0.06 ^d^	3.55 ± 0.06 ^a^	2.85 ± 0.08 ^c^	3.10 ± 0.09 ^b^	3.10 ± 0.10 ^b^
His	1.00 ± 0.01 ^b^	0.62 ± 0.03 ^c^	1.03 ± 0.03 ^b^	1.02 ± 0.04 ^b^	1.13 ± 0.04 ^ab^	1.29 ± 0.20 ^a^
Arg	3.78 ± 0.13 ^d^	1.74 ± 0.08 ^e^	5.10 ± 0.03 ^a^	4.14 ± 0.12 ^c^	4.42 ± 0.14 ^b^	4.37 ± 0.10 ^bc^
Pro *	3.30 ± 0.09 ^c^	2.21 ± 0.05 ^e^	2.80 ± 0.07 ^d^	4.90 ± 0.08 ^a^	3.89 ± 0.10 ^b^	5.06 ± 0.07 ^a^
AAA	4.11 ± 0.37 ^c^	2.28 ± 0.10 ^d^	5.01 ± 0.10 ^a^	4.48 ± 0.14 ^bc^	4.71 ± 0.09 ^ab^	4.86 ± 0.10 ^ab^
BCAA	6.39 ± 0.23 ^b^	3.44 ± 0.36 ^d^	5.88 ± 0.12 ^c^	6.25 ± 0.10 ^bc^	6.63 ± 0.09 ^b^	7.52 ± 0.07 ^a^
HAA	17.49 ± 0.78 ^c^	10.08 ± 0.38 ^d^	18.38 ± 0.19 ^c^	20.40 ± 0.15 ^b^	20.06 ± 0.42 ^b^	22.70 ± 0.14 ^a^
TAA	48.75 ± 2.71 ^c^	29.49 ± 0.90 ^d^	50.91 ± 0.53 ^c^	61.34 ± 0.44 ^a^	56.72 ± 1.45 ^b^	64.01 ± 1.31 ^a^

Values are expressed as mean ± SD (*n* = 3). a, b, c, d, and e in the rows indicate differences between groups (*p* < 0.05). * Hydrophobic amino acid (HAA); ^#^ essential amino acid (EAA); ^1^ branched-chain amino acid (BCAA); ^2^ aromatic amino acid (AAA); total amino acid (TAA).

**Table 2:** Virtual prediction of toxicity and ACEI activity of seventeen peptides.

Number	Peptide Sequence	Molecular Weight	AHT-SVM	Toxin
1	CDFEIQFE	1086.4328	−1.39	Non-Toxin
2	EKPDFGK	819.4126	−0.44	Non-Toxin
3	EQPALGK	741.4021	−0.53	Non-Toxin
4	FHGLPAK	768.4282	1.38	Non-Toxin
5	NALRTAM	775.4010	−2.11	Non-Toxin
6	RGLPAYE	804.4130	0.67	Non-Toxin
7	TLRVDIK	843.5178	−1.1	Non-Toxin
8	VPSDVEF	791.3701	1.17	Non-Toxin
9	YTDANGE	768.2926	−1.27	Non-Toxin
10	LIGGHQK	751.4340	−0.69	Non-Toxin
11	PWHFDRNY	1133.5043	−0.27	Non-Toxin
12	IIAPPERKY	1085.6233	1.51	Non-Toxin
13	LRVAPEE	812.4392	−0.01	Non-Toxin
14	WWNTSNIY	1082.4821	−0.2	Non-Toxin
15	HYDRYYF	1062.4559	−0.19	Non-Toxin
16	SARVDGK	731.3926	−2.02	Non-Toxin
17	VLHTLGF	785.4435	−0.96	Non-Toxin

**Table 3:** Elution condition parameters.

Time (min)	Liquid A	Liquid B
0.0	98%	2%
10.0	92%	8%
55.0	73%	27%
65.0	63%	37%
70.0	2%	98%
75.0	2%	98%
78.0	95%	5%
81.0	95%	5%
85	2%	98%
90	2%	98%

**Table 4:** Mass spectrometry conditions.

Parameter Name	Parameter Value
Ion mode	Positive ion mode
Primary scanning range	350–1500 Da
Secondary scanning range	Automatic control based on parent ion mass-to-charge ratio
Capillary temperature	320 °C
Ion source spray voltage	2200 V
Fragmentation conditions	HCD

**Table 5:** The screening parameters.

Parameter Name	Parameter Value
The mass range of the parent ion	350–1500 Da
The minimum number of peaks in the secondary mass spectra	10
S/N threshold	1.5

## Data Availability

The original data presented in the study are included in the article; further inquiries can be directed to the corresponding author.

## Supplementary Materials

The following supporting information can be downloaded at: https://www.mdpi.com/article/10.3390/md23020081/s1, Figure S1. Frequency of amino acid occurrence at the N-terminal (a) and C-terminal (b) of the peptide chain of the recorded ACEI peptide. Figure S2. (a) to (d) are 2D diagrams of the molecular docking results of FHGLPAK, IIAPPERKY, RGLPAYE, VPSDVEF with ACE. In 2D diagrams, the green region indicates hydrogen bond interaction, orange region is salt bridge, pink and purple regions are hydrophobic interactions.
