# Supplementary material for: Screening of Potential Angiotensin-Converting Enzyme-Inhibitory Peptides in Squid (Todarodes pacificus) Skin Hydrolysates: Preliminary Study of Its Mechanism of Inhibition
Source: Mar Drugs. 2025 Feb 13;23(2):81. doi: 10.3390/md23020081 (PMC11857160; doi:10.3390/md23020081)
Supplement: Supplementary file 1 [file marinedrugs-23-00081-s001.zip › marinedrugs-3420072-supplementary.pdf]

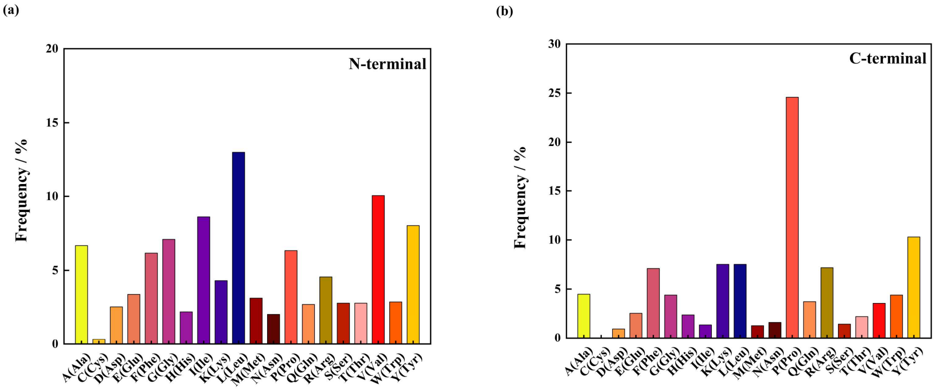

Figure S1. Frequency of amino acid occurrence at the N-terminal (a) and C-terminal (b) of the peptide chain of the recorded ACEI peptide.

(a)

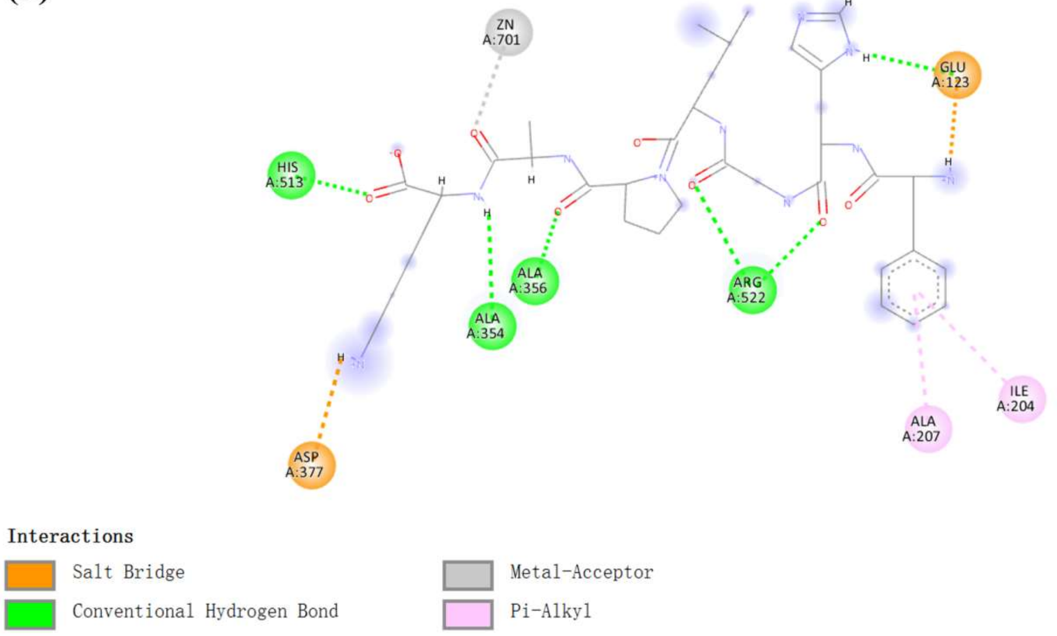

(b)

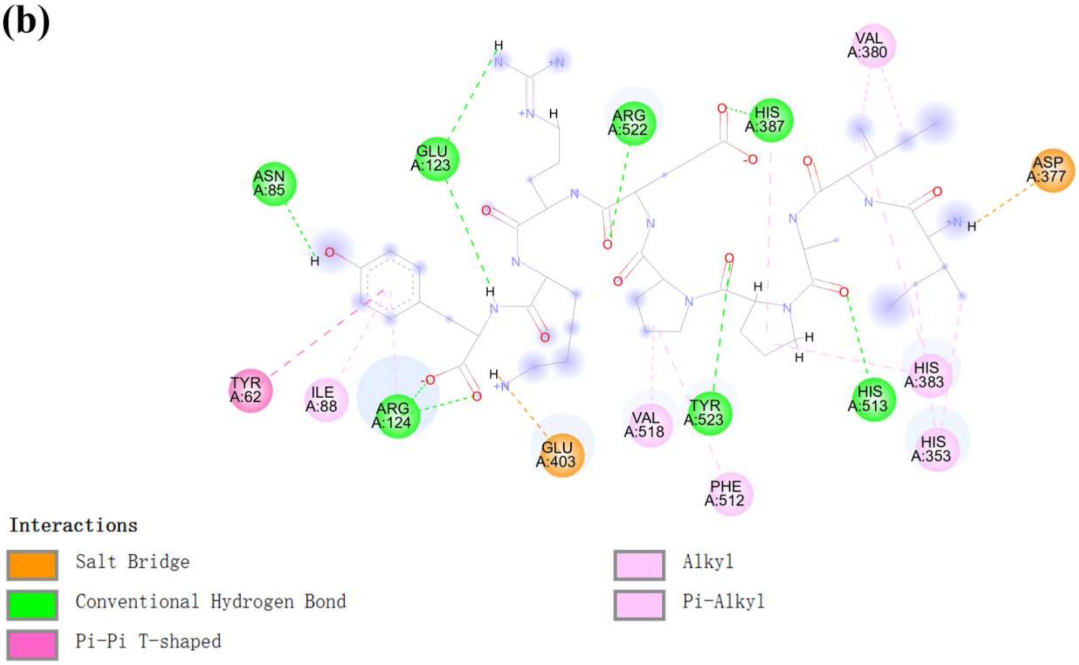
VAL A:380
ARG A:522
HIS A:387
GLU A:123
ASN A:85
ASP A:377
TYR A:62
ILE A:88
ARG A:124
GLU A:403
VAL A:518
TYR A:523
PHE A:512
HIS A:513
HIS A:383
HIS A:353
Interactions
Salt Bridge
Conventional Hydrogen Bond
Pi-Pi T-shaped
Alkyl
Pi-Alkyl

(c)

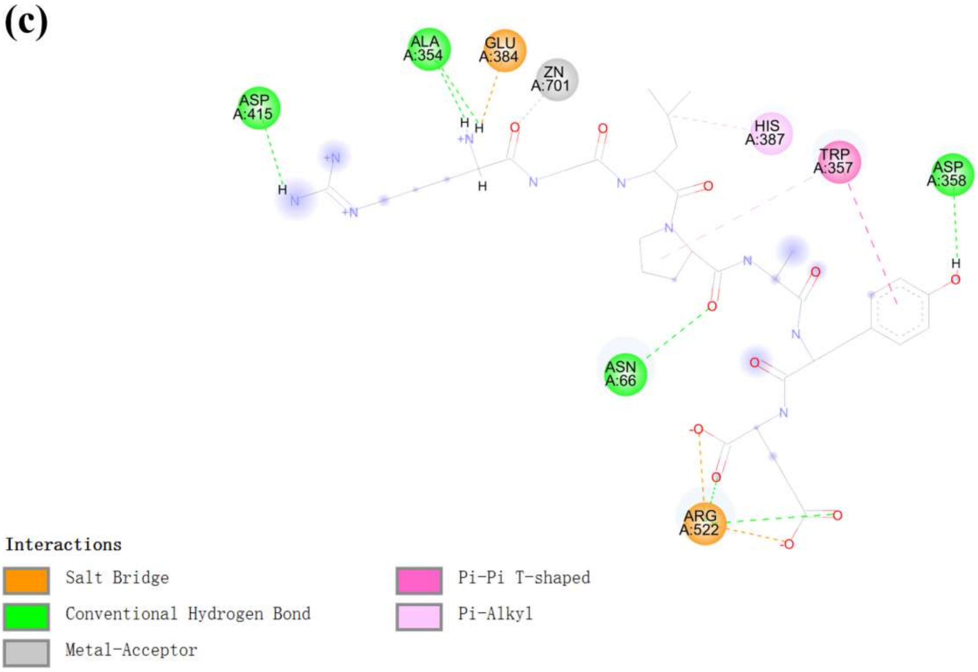
ALA A:354
GLU A:384
ZN A:701
ASP A:415
HIS A:387
TRP A:357
ASP A:358
ASN A:66
ARG A:522
Interactions
Salt Bridge
Conventional Hydrogen Bond
Metal-Acceptor
Pi-Pi T-shaped
Pi-Alkyl

(d)

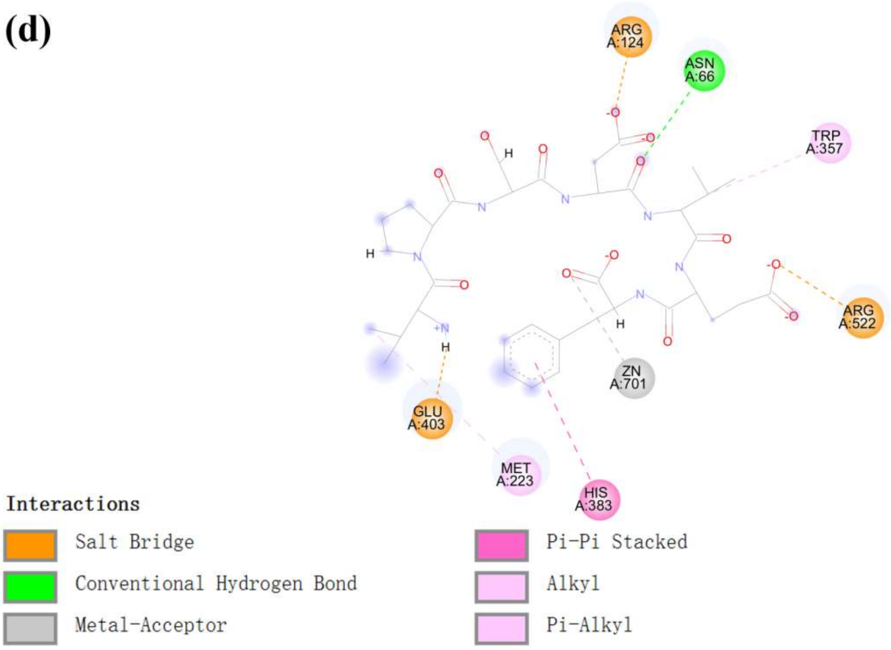

Figure S2. (a) to (d) are 2D diagrams of the molecular docking results of FHGLPAK, IIAPPERKY, RGLPAYE, VPSDVEF with ACE. In 2D diagrams, the green region indicates hydrogen bond interaction, orange region is salt bridge, pink and purple regions are hydrophobic interactions.
